# Host species and environment drivers of ectoparasite community of rodents in a Mojave Desert wetlands

**DOI:** 10.1371/journal.pone.0269160

**Published:** 2022-06-02

**Authors:** Andrés M. López-Pérez, Risa Pesapane, Deana L. Clifford, Laura Backus, Patrick Foley, Ashley Voll, Ricardo Bassini Silva, Janet Foley

**Affiliations:** 1 Department of Medicine and Epidemiology, School of Veterinary Medicine, University of California, Davis, California, United States of America; 2 Department of Veterinary Preventive Medicine, School of Environment and Natural Resources, The Ohio State University, Columbus, Ohio, United States of America; 3 Wildlife Investigations Lab, California Department of Fish and Wildlife, Rancho Cordova, California, United States of America; 4 Department of Biological Sciences, California State University Sacramento, Sacramento, California, United States of America; 5 Zoological Collections Laboratory, Butantan Institute, Butantã, São Paulo, São Paulo, Brazil; 6 Faculty of Agrarian and Veterinary Sciences-UNESP, Department of Pathology, Reproduction and Unique Health, Jaboticabal, São Paulo, Brazil; University of Pretoria, SOUTH AFRICA

## Abstract

Drivers of patterns of ectoparasitism in rodents in patchy Mojave Desert wetlands were investigated. A total of 1,571 ectoparasites in Mesostigmata, Trombidiformes, Siphonaptera and Ixodida were collected from 341 rodents (*Microtus californicus scirpensis*, *Mus musculus*, *Reithrodontomys megalotis*, *Peromyscus eremicus*, and *Neotoma lepida*) at eleven marshes. Trombiculids accounted for 82.5% of mites, followed by the mesostigmatid *Ornithonyssus bacoti* (17.5%), with chiggers predominating on voles and harvest mice. There were at least three genera of chiggers (*Eutrombicula alfreddugesi*, *Euschoengastia* sp. novel, and *Blankaartia* sp. novel). Fleas included *Orchopeas leucopus* (90.3% of all fleas) and *O*. *sexdentatus* (9.7%), and ticks were the novel endemic *Ixodes mojavensis* (82.1% of ticks) and *Dermacentor similis* (17.9%). On all hosts and at all marshes, coverage-based rarefaction sampling was over 96%, indicating coverage sufficient for analysis. Dissimilarities in ectoparasite community structure were driven mainly by chiggers, *I*. *mojavensis* and *O*. *leucopus*. Northern marshes were dominated by chiggers; central marshes by *I*. *mojavensis*; and southern marshes by *O*. *leucopus*. Primary determinants of ectoparasite community structure were host species, patch size, and parasite interspecific interactions. Host species richness and environmental factors such as patch distance and water and plant availability were not significantly associated with patterns of ectoparasitism. There were nine (60%) significant negative pairwise associations between ectoparasite taxa and no significant positive relationships. *Ixodes mojavensis* had the highest number of negative associations (with five other species), followed by chiggers and *O*. *bacoti* with two negative associations each. The study area is among the most arid in North America and supports numerous rare and endemic species in increasingly isolated wetland habitat patches; knowledge of ectoparasite ecology in this region identifies potential ectoparasite vectors, and provides information needed to design and implement programs to manage vector-borne diseases for purposes of wildlife conservation.

## Introduction

Ectoparasites are a taxonomically diverse group including arthropods such as ticks, fleas, and mites on the exterior integument of humans and other animals [1]. Infestation by ectoparasites can reduce host fitness directly (e.g. by causing death or reducing reproduction) and allow for transmission of pathogens [2–4]. Vector-borne diseases are among the most concerning of infectious diseases worldwide [5], often with rodents playing key roles in their ecology, as reservoirs, hosts for vectors, or both [6–9]. Some vector-borne disease, such as plague and tularemia, can cause death in the rodents themselves [6,10,11]. Understanding factors that influence geographical and host distributions of ectoparasites has important implications for infectious disease control and human and animal health [12].

Spatial distributions and community structure of ectoparasites are influenced by numerous biotic and abiotic factors [13–15]. Important biotic factors include host species, sex, age, body condition, size, and immune status, host population size (abundance and distribution), and host community diversity [14,16,17]. In addition, as hosts may be infested with more than one ectoparasite species, parasites co-infesting a host individual might experience interspecific interactions including antagonism or facilitation [18,19]. Antagonistic interactions may be directly mediated by physical or chemical signals or indirectly via resource competition (bottom-up regulation). Facilitation is primarily mediated by indirect mechanisms such as host immune suppression (top-down regulation). Facilitative and competitive interactions have been mainly studied in endoparasites and less so in ectoparasites [20–22], with exceptions including other studies in rodents which found apparent competitive exclusion among chiggers, ticks, fleas, and lice [23,24]; among fleas, chiggers, and cuterebrid botflies [25]; and among different flea species [26,27].

Among ectoparasites such as ticks and fleas that spend much of their lives off-host [28], spatial distribution and community structure are also influenced by abiotic factors such as seasonal variation in temperature and precipitation [29–32]. Moreover, human disturbance [33] and geographical distance among host populations (so-called host connectivity) can also influence the spread and establishment of parasites [34–36]. When hosts are distributed among spatially disjunct patches, those habitat and host-specific factors that influence dispersal among patches also influence the permeability of ectoparasites into patches [37,38].

Environment and host factors as drivers of spatial patterns and community of ectoparasites have been poorly studied in rodents in North America, focused either on one host species or population. For example, a study in Kentucky found impacts of host age and sex, site, and season on mite, tick and flea infestations [39]. The flea community on two chipmunk hosts in Yosemite, California was predicted by host sex and temperature/elevation [40].

This study examined biotic and abiotic drivers of patterns of ectoparasitism in a community of rodents distributed among patchy wetlands in the central Mojave Desert in California. This area is one of the most arid in North America [41] and supports numerous rare and endemic species in increasingly isolated wetland habitat patches, such as the critically endangered Amargosa vole (*Microtus californicus scirpensis*). Ongoing habitat degradation and loss of water driven by anthropogenic hydrologic alterations (e.g. ground-water pumping and land clearing) and climate change [42,43] may have considerable impacts on ecological interactions among hosts and ectoparasites and vector-borne diseases in this region.

The aim of this study was to determine how host individuals, species, and community interact with environment factors to influence rodent ectoparasite community structure in wetlands in the Mojave Desert. We differentiated between ectoparasite fauna on host individuals and “component communities” which are those parasites on host individuals of a particular population, and performed four analyses. First, we calculated parasite abundance and diversity by host species and marsh patch. Secondly, we represented parasite community composition by host species and patch using non-metric multidimensional scaling and then examined for significant differences using PerMANOVA, identifying parasite species most responsible for the differences using crossed similarity percentage analysis. Thirdly, we tested for significant positive and negative co-occurrence of parasite species on hosts and in marshes. Lastly, we developed multivariate random forests to evaluate influences of host species and environment factors on ectoparasite community structure. Given that parasite dispersal and host-parasite coevolution are influenced by host range which impacts parasite habitat and host-linked dispersion [44], we hypothesized that structure of the ectoparasite community in Mojave Desert wetlands would be most strongly influenced by host identity. We also hypothesized that the size of discrete marsh patches and distance among patches would affect the community of ectoparasites, since smaller habitat fragments may have fewer species due to isolation and being too small for persistence [45].

## Materials and methods

### Study area

**Fig 1:**
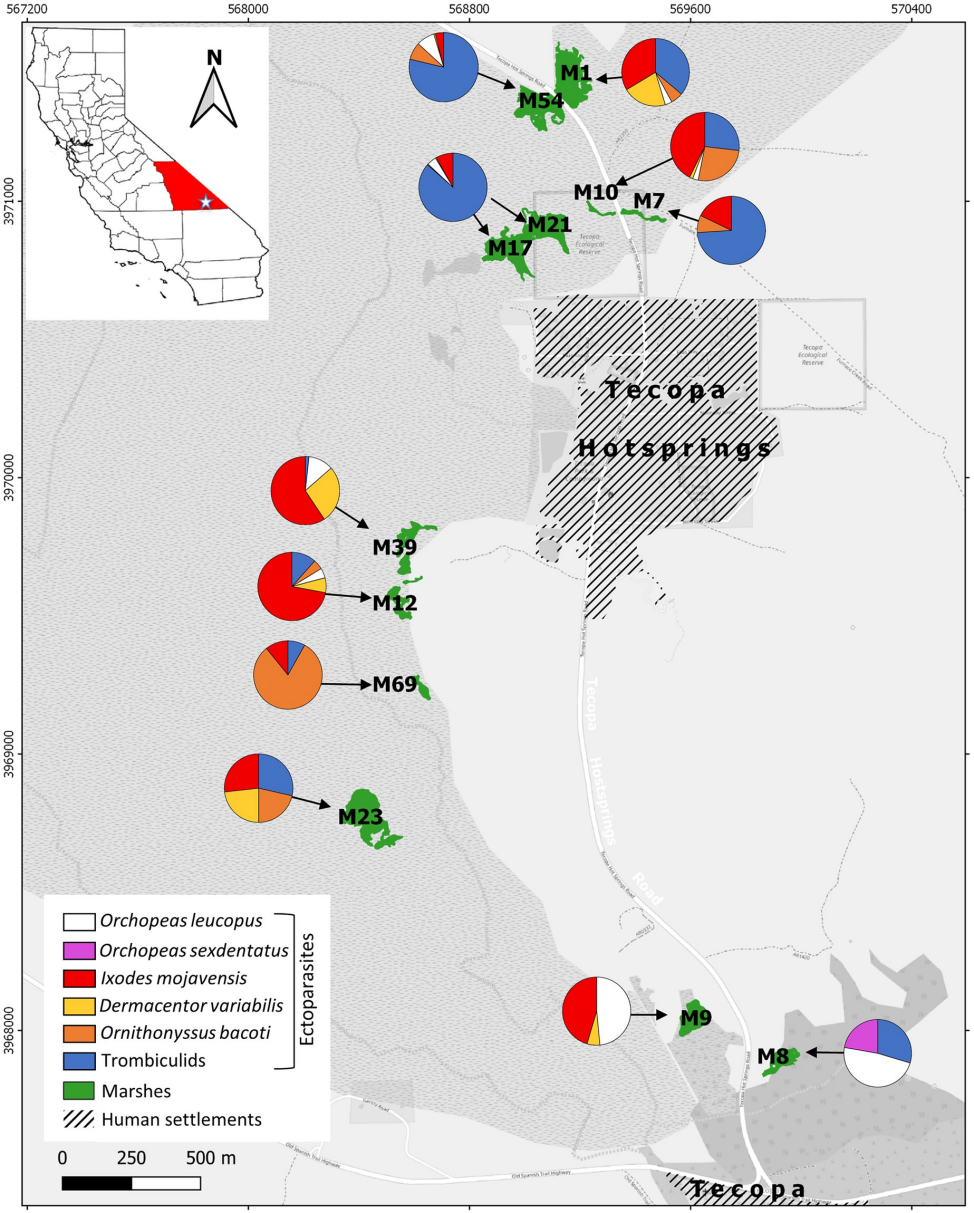
Map of Tecopa, California in the Mojave Desert showing pie charts of community composition of ectoparasites on rodents for eleven marsh habitat patches surveyed. Marshes are numbered according to conventions of the ad hoc Amargosa vole team. Reprinted from the OpenStreetMap vector basemap hosted by Environmental Systems Research Institute (Esri) and provided under a CC BY 4.0 license, (Map data © OpenStreetMap contributors, Map layer by Esri 2019).

Rodents were live-trapped in Tecopa within the Amargosa River basin in the Mojave Desert in southeastern Inyo County, California, USA (35.8752 N, -116.2343 E, Fig 1). The elevation ranges from 390–417 m and the local climate is arid and variable, with annual mean rainfall of 12.3 cm, and mean temperature ranges from 41.4 °C in summer to 3.2 °C in winter. The Amargosa River in this region is often subterranean, although ephemeral and spring-fed perennial surface flows support marsh habitat. Riparian vegetation predominantly consists of bulrush (*Schoenoplectus americanus*) with a mix of desert salt grass (*Distichlis spicata*), rushes (*Juncus cooperi* and *J*. *balticus*), and mixed herb communities including yerba mansa (*Anemopsis californica*) and western reed (*Phragmites australis*).

### Trapping localities and sampling

Rodent trapping and sampling were conducted during all four seasons of the year at 12 marshes from 2011 to 2019, although sampling occurred when management and recovery actions dictated trapping and was not conducted at systematic intervals. Marshes 17 and 21 were grouped together for analysis (Fig 1). Each of the marshes was surveyed over 3–6 consecutive days along pre-established transects using Sherman live traps (7.6 cm x 8.9 cm x 22.9 cm; HB Sherman, Tallahassee, FL, USA). Numbers of traps varied according to the size of habitat fragment, from 30 to 108 traps per transect. The traps were baited with a mix of peanut butter, horse feed (corn, barley, oats, and wheat with molasses), and apples or peanut butter and oats. Baited traps were placed under vegetation, kept open overnight, and then checked before dawn. We recorded locations of traps using handheld global positioning system (GPS) receivers (Garmin 62S GPS, Garmin International, Olathe, KS, USA). Each individual was processed in a separate clean plastic bag, and identified to species, sex, and age class (juvenile, subadult, and adult). In addition, animals were ear tagged (model 1005–1; National Band and Tag Co., Newport, KY, USA) and weighed. Each animal was inspected systematically for ectoparasites, all of which were removed with forceps and placed into microtubes containing 70% ethanol. After handling, all animals were released at their site of capture.

All procedures for trapping and handling rodents followed the guidelines of the American Society of Mammalogists [46] and were approved by the US Fish and Wildlife Service (USFWS) under Recovery Permit #TE546414A-2, California Department of Fish & Wildlife (CDFW), Bureau of Land Management (BLM), and UC Davis Institutional Animal Care and Use Committee.

### Arthropod identification

In order to visualize structures required for flea identification, we cleared their exoskeletons by incubating in dilute KOH for 24 hr, and then dehydrating in an ethanol series (75, 85, 95, and 100% for 30 min each). Fleas were mounted individually on slides with Euparal (BioQuip, Rancho Dominguez, CA, USA) for examination using a stereo microscope, and identified using taxonomic keys [47–49]. Ticks were identified morphologically using dichotomous keys [50–52]. To confirm identity of the single species of *Dermacentor* tick detected, a 280 base pair segment of the 16S rDNA mitochondrial gene was sequenced for three individual ticks following [53]. Sequences were submitted to Genbank (accession numbers are: MZ996569, MZ996570, and MZ996571) and compared with existing *Dermacentor* spp. 16S sequences. Phylogenetic analysis was performed comparing our sequences to multiple *D*. *variabilis* accessions each from western North America and eastern North America using MEGA X [54].

Individual chiggers were gently separated and then cleared, stained, and mounted on slides using polyvinyl alcohol (PVA) Mounting Medium with Double Stain (BioQuip, Rancho Dominguez, CA) as follows. In a 1.5mL centrifuge tube, one drop of stain was added to 1mL of the all-in-one clearing and mounting medium and then mixed thoroughly. Chiggers were incubated in 2–3 drops of this mixture at 48-52C for 45–60 minutes before being slide-mounted. Larger mites were cleared and mounted using the same method as described for fleas. All mites were examined using a compound microscope, and identified using taxonomic keys [55–59].

### Landscape features

We defined habitat as dominated by bulrush using a vegetation cover layer provided by the United States Geological Survey (USGS). For each of the 11 marshes, we calculated total area and perimeter, distance to the nearest marshes, and distance to the nearest human settlement using QGIS (www.qGIS.org) functions. We obtained plant species richness from earlier surveys [60] and calculated mean water depth from measurements collected systematically every 20m throughout bulrush-dominated portions of each marsh. Water depth measurements were collected twice (peak winter and summer) during 2017 to capture seasonal changes in water levels although overall water levels could change annually as well, particularly under impacts of drought.

### Statistical analysis

#### Ectoparasite richness and community dissimilarity (diversity analysis)

Data were managed in Excel (Microsoft, Redmond, WA) and analyzed in the online freeware application iNEXT [61] and in R (R Development Core Team., 2008) via R Studio (RStudio Team, 2015). Statistical significance was inferred if p ≤ 0.05. For each patch and rodent host species, we calculated ectoparasite abundance and diversity. We developed coverage-based rarefaction and extrapolation curves to compute diversity estimates to evaluate sampling completeness of ectoparasite community using iNEXT (iNterpolation and EXTrapolation). This procedure ensures that differences in ectoparasite richness were not driven by imbalances in sample coverage for rodent host and sampling locations. We calculated interpolation and extrapolation sampling curves of Hill numbers for the species richness (q = 0) and Shannon diversity (q = 1) for observed individual-based abundance data of ectoparasites. Diversity estimates and related statistics were computed for 100 bootstraps and 95% confidence intervals for each diversity and sample coverage estimate.

In order to represent differences in ectoparasite community composition among patches and host species, we performed non-metric multidimensional scaling (NMDS) in R package "vegan" [62]. Communities were analyzed at two levels of organizations: infracommunity and component community. Infracommunities include all parasite populations on an individual host, while component communities include all infracommunities within the same host population. NMDS is a rank-based approach that ordinates and represents pairwise dissimilarity among ectoparasite communities using a low-dimensional space. All NMDS analyses were conducted using Bray–Curtis dissimilarities using untransformed ectoparasite species abundances at the infracommunity scale (S1 Table). We calculated the stress values generated by the algorithm as quantitative measure of ordination fit using Kruskal’s stress, S [63]. S < 0. 0.1 corresponds to a good fit; S < 0.2 can still be usable, although values > 0.2 are considered suspect [63]. In the NMDS for component communities, we calculated the capture rate for each community by dividing the abundance of ectoparasites by the number of host individuals captured (sampling effort) to account for the effect of high abundance parasite taxa and variation in number of individuals ectoparasites per host species. To determine whether there were significant differences in parasite component community structure hosts and marshes, we performed a permutational multivariate ANOVA (PerMANOVA), using site as a factor and 999 permutations. In addition, we performed a 2-way crossed similarity percentage (SIMPER) analysis to identify the parasite species most responsible for the variation in parasite community structure among sites and host species.

#### Ectoparasite species co-occurrence

We assessed co-occurrence patterns of ectoparasite species pairs on host individuals and by marsh. A probabilistic model of species co-occurrence [64] implemented in the “cooccur” package [65] was applied to test for statistically significant pair-wise patterns of species co-occurrence. The model determines whether any two ectoparasite species were more or less likely relative to chance to co-occur if the two species were distributed independently of one another among a set of sites. Observed co-occurrence is compared to the expected co-occurrence where the latter is the product of the probability of occurrence of the two species multiplied by the number of sampling sites: E(N1,2) = P(1)×P(2)×N. The model determines the probability that the observed frequency of co-occurrence is significantly large and greater than expected (positive association), significantly small and less than expected (negative association), or not significantly different and approximately equal to expected (random association) [64]. In this study, the model was implemented using a co-occurrence matrix where species were ectoparasites and sites represented host individuals. Pairs of species with expected co-occurrence < 1 were removed from the analysis.

#### Impacts of biotic and abiotic factors on ectoparasite community

We performed a multivariate random forest (MRF) model to determine how host species and environmental factors influenced the composition of ectoparasite infracommunity and component community. Response variables were the abundances of each ectoparasite species. Predictor variables were season, total marsh area and perimeter, minimum distance to the nearest marshes and to the nearest human settlement, water depth, plant species richness, host sex and age, host species, and host species richness. We performed two MRFs, examining predictor variables’ effects on parasite communities at the infracommunity and component community scale. Sample sizes limited inclusion of season, host sex, and host age as predictor variables to the MRF model at the infracommunity scale.

The MRF model built an ensemble of several hundred trees using bootstrapped subsamples of the original data and aggregating the results [66]. The prediction error for each tree was calculated and the prediction error for the forest was the average prediction error of the individual trees. MRFs were built using 1000 trees, and "Breiman-Cutler" variable importance was calculated by randomly permuting the values of the variables, running them through the model, and evaluating the change in the mean squared error (MSE). In these variable importance permutations, each predictor variable was placed in the out-of-sample (out-of-bag for univariate analyses) data for the tree model. The out-of-sample prediction error was then calculated with and without the permutation and averaged over the MRF trees. Greater differences between the permuted and non-permuted out-of-sample prediction error imply greater variable importance. MRF models were done using R package randomForestSRC [67].

We also calculated the Spearman’s rank correlation coefficient to identify associations between the water depth of marshes and the abundance of ectoparasite species in rodents.

## Results

### Rodents and ectoparasite community structure

A total of 341 rodents were infested with at least one ectoparasite (mite, flea, or tick), including 262 Amargosa voles (76.8% of all infested rodents), 44 western harvest mice (*Reithrodontomys megalotis*, 12.9%), 18 house mice (*Mus musculus*, 5.3%), 10 cactus mice (*Peromyscus eremicus*, 2.9%), and seven desert woodrats (*Neotoma lepida*, 2.0%, Table 1).

**Table 1:** Number of ectoparasites collected from rodents from 11 marshes in Tecopa, California, US between 2011 and 2019.

Host species	Number of hosts	Mites		Fleas		Ticks	
		Chiggers	*Ornithonyssus bacoti*	*Orchopeas leucopus*	*Orchopeas sexdentatus*	*Dermacentor similis*	*Ixodes mojavensis*
*Microtus californicus scirpensis*	262	788	184	52	0	56	286
*Mus musculus*	18	13	1	11	0	0	4
*Neotoma lepida*	7	9	0	0	11	0	0
*Peromyscus eremicus*	10	6	0	21	1	0	0
*Reithrodontomys megalotis*	44	73	4	28	0	10	13
Total	341	889	189	112	12	66	303

Of all rodents evaluated, mites were observed on 126 (36.5%), fleas on 74 (21.4%), and ticks on 178 (51.6%). In total, 1,571 ectoparasites were collected, representing four orders (Mesostigmata, Trombidiformes, Ixodida, and Siphonaptera, Table 1).

**Fig 2:**
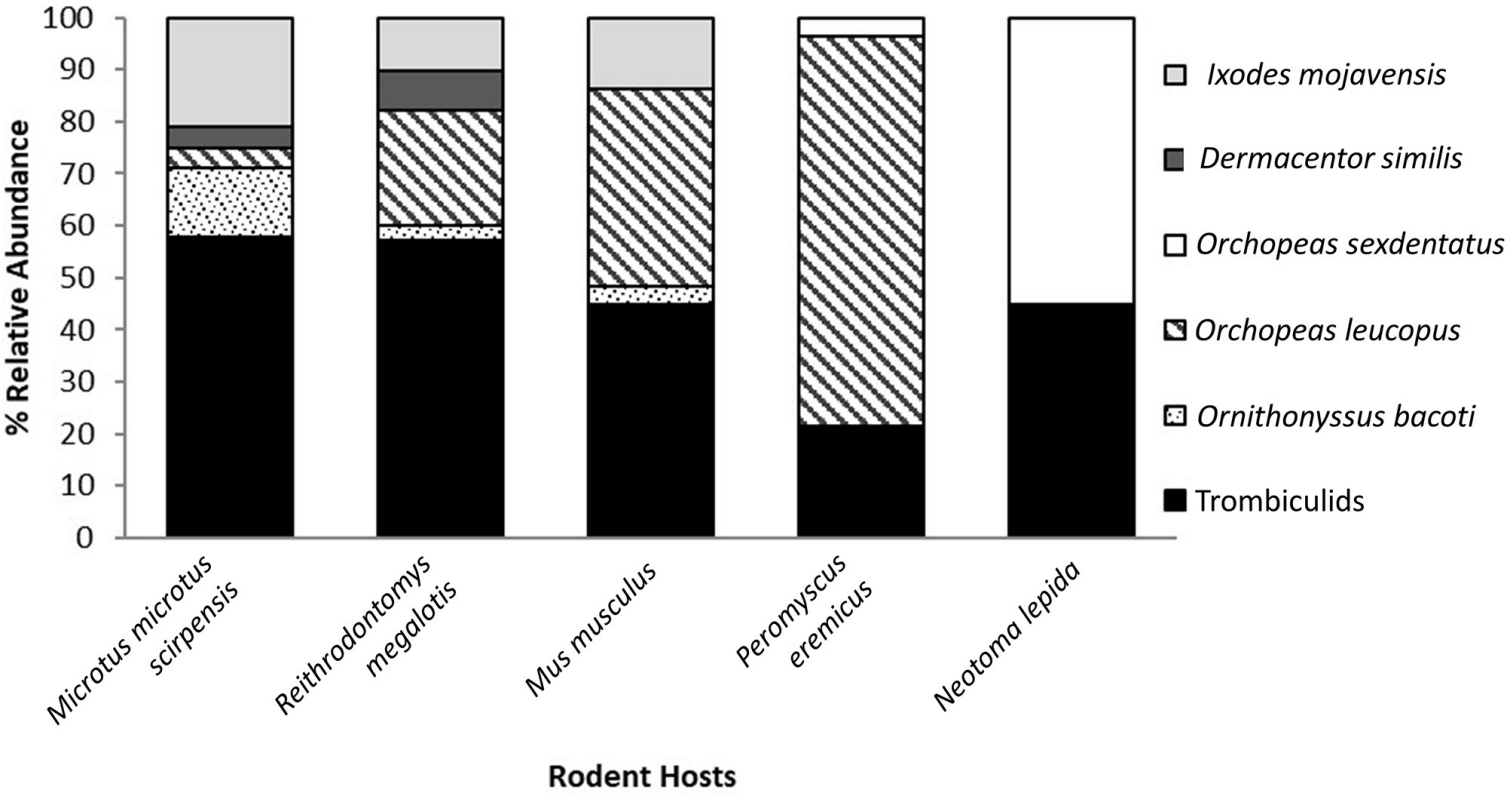
Distribution of rodent ectoparasite taxa among marshes near Tecopa, California, US.

There were 1,078 individual mesostigmatid and chigger mites collected from 101 Amargosa voles, 13 western harvest mice, seven house mice, four woodrats, and one cactus mouse (Fig 2). Chiggers accounted for 82.5% of all mites, followed by the mesostigmatid *Ornithonyssus bacoti* (17.5%), with chiggers comprising the majority of the ectoparasite fauna on voles and harvest mice and plurality on house mice and woodrats. We were able to identify at least three genera of chiggers (*Eutrombicula alfreddugesi*, *Euschoengastia* sp. novel, and *Blankaartia* sp. novel). All *O*. *bacoti* individuals were post-larval stage. One hundred and twenty-four fleas belonging to two species were collected from 31 Amargosa voles, 18 western harvest mice, 10 cactus mice, 10 house mice, and five woodrats (Table 1). The flea community was dominated by *Orchopeas leucopus* (90.3% of all fleas collected and the majority of all ectoparasites on cactus mice), followed by *O*. *sexdentatus* (9.7% of all fleas collected and the majority of all ectoparasites on woodrats). There were 369 ticks belonging to two species collected from 157 Amargosa voles, 18 western harvest mice, and three house mice. The most abundant tick was *Ixodes mojavensis* (82.1% of all ticks collected) followed by *Dermacentor similis* (17.9%). Given the recent re-description of western *D*. *variabilis* as the separate species *D*. *similis* [68], we confirmed the identification of the Amargosa ticks with a BLAST search of the GenBank database. Our three sequenced samples had 100% identity to multiple *D*. *variabilis* submissions, all from the western United States. On phylogenetic analysis, our samples clustered closely with only the western North American sequences. and as a separate clade from eastern, true *D*. *variabilis*. *Ixodes mojavensis* predominated on voles and house mice, while *D*. *similis* was only found on voles and harvest mice. Northern marshes (M1, M7, M54, M17-21) tended to be dominated by chiggers with relative abundances ranging from 36 to 86% of all ectoparasites; central marshes (M12, M39, M69) were dominated by *I*. *mojavensis* ticks (26–72% of all ectoparasites), and southern marshes (M8, M9) were dominated by *O*. *leucopus* fleas (48 to 49% of all ectoparasites, Fig 1). On all hosts and at all marshes, the coverage-based rarefaction sampling was over 96%, indicating coverage of the sampled ectoparasite communities sufficient for analysis.

**Fig 3:**
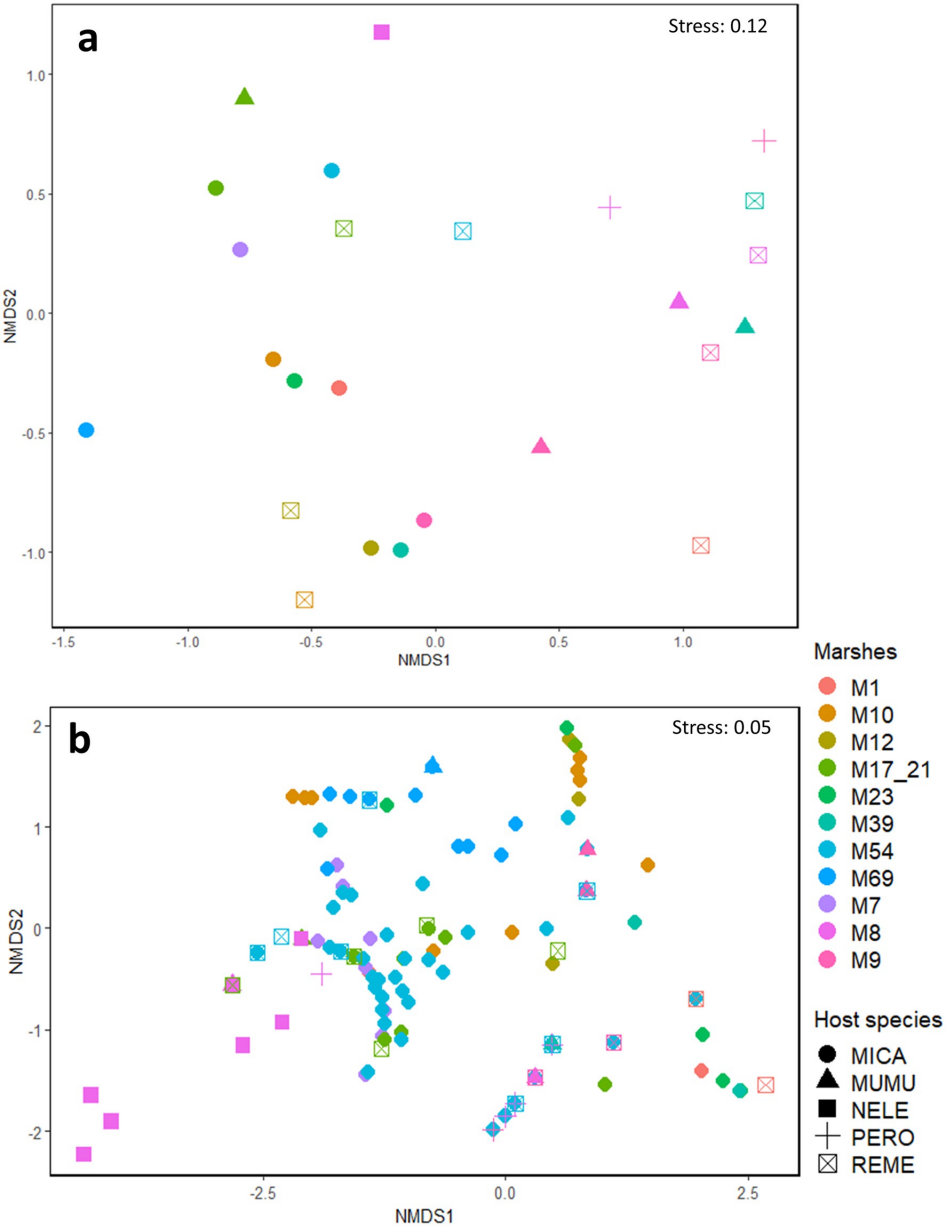
Non-metric multidimensional scaling (NMDS) plots of ectoparasite community. Ectoparasite component communities (3a) and infracommunities (3b) of rodents from 11 marsh patches in Tecopa, California, US. Host species: MICA, *Microtus californicus scirpensis*; MUMU, *Mus musculus*; NELE, *Neotoma lepida*; PERO, *Peromyscus eremicus*; REME, *Reithrodontomys megalotis*.

NMDS (Fig 3a and 3b) and PERMANOVA analyses showed that infracommunity and component community composition differed among marshes (F = 8.039, df = 10, P = 0.001 and F = 2.028, df = 10, P = 0.015, respectively) and hosts (F = 4.323, df = 4, P = 0.001 and F = 2.449, df = 4, P = 0.005, respectively). The observed dissimilarities in ectoparasite component community structure were driven mainly by chiggers and *O*. *leucopus* fleas either among all marshes or all host rodent species. At the infracommunity scale, chiggers and *I*. *mojavensis* ticks were the ectoparasite species that contributed more to the dissimilarities among all marshes. However, a few marshes (Marshes 8, 23, and 69) had unique patterns of dissimilarities in both infra and component ectoparasite community structures driven by *O*. *leucopus*, *D*. *similis*, and *O*. *bacoti*, respectively. Finally, observed differences among Amargosa voles, house mice, and harvest mice were driven by chiggers and *I*. *mojavensis* ticks, whereas differences between cactus mice and other rodents were driven by *O*. *leucopus* and differences between desert woodrat and other rodent species were driven mainly by *O*. *sexdentatus*.

### Co-occurrence among ectoparasite species

**Fig 4:**
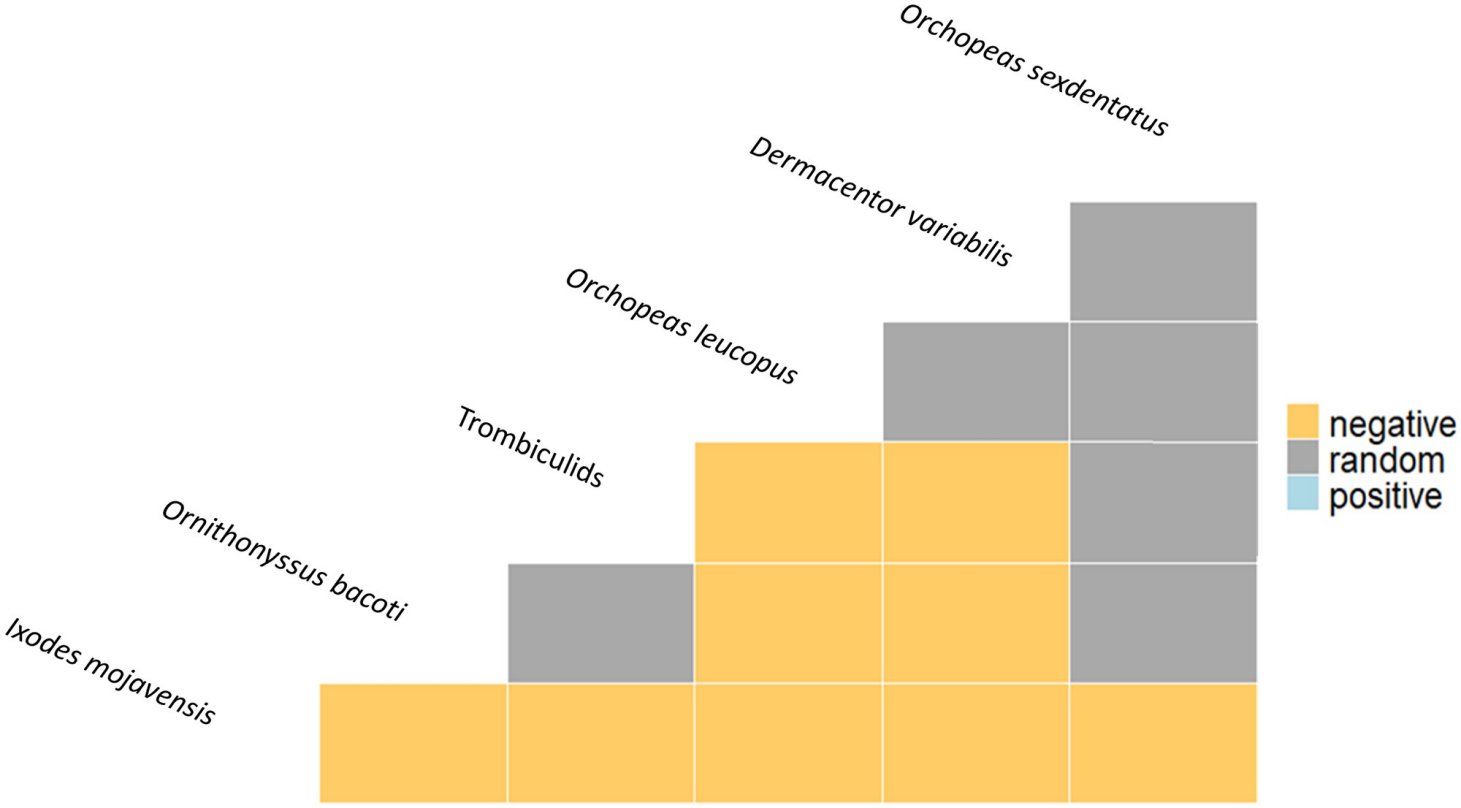
Taxon co-occurrence matrix for all possible pairwise comparisons between mites, fleas and ticks collected from rodents in Tecopa, California, US.

Out of the 15 ectoparasite species pair combinations that we studied in rodents in the Mojave Desert, there were nine (60%) significant negative associations, with no significant positive relationships (Fig 4). *Ixodes mojavensis* had the highest number of negative significant associations with five other species, followed by chiggers and *O*. *bacoti* with two negative associations each.

### Host and landscape predictors of ectoparasite community

**Fig 5:**
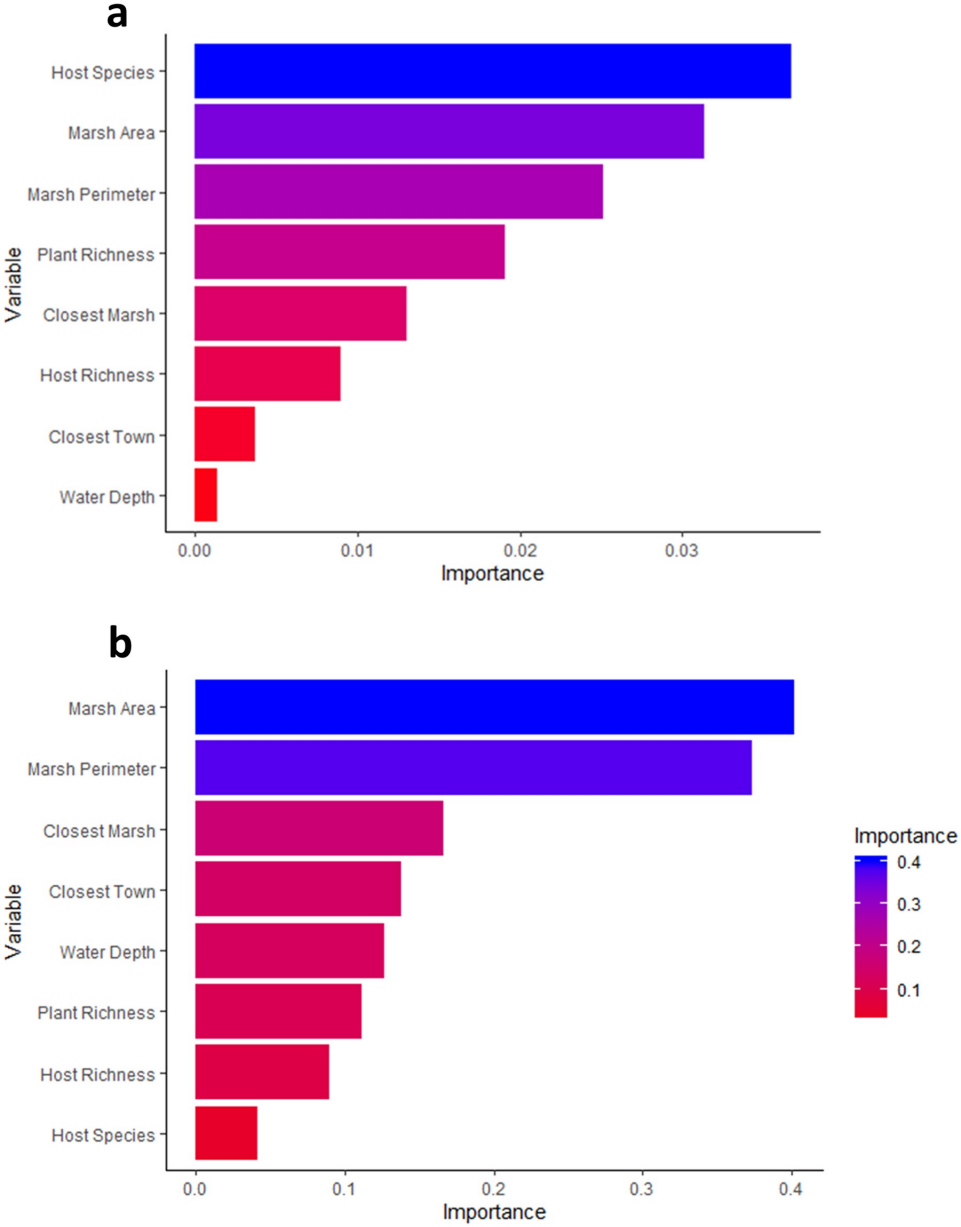
Multivariate random forest model and relative importance of predictor variables of ectoparasite community. Ectoparasite component communities (5a) and infracommunities (5b) of rodents from 11 marsh patches in Tecopa, California, US.

Landscape characteristics of the marshes and numbers of parasitized rodent hosts in each marsh are summarized in Table 2. Due to the fact that season, host sex and host age were not important predictors for the structure of ectoparasite infracommunity, we removed those three variables from the overall model. The MRF model accounted for 20.7% of the variance in the structure of ectoparasite component community, with host species and area and perimeter of the marshes being the most important predictors (Fig 5a). On the other hand, the model predicting ectoparasite community structure at the infracommunity scale explained 10.3% variance, with area and perimeter of the marshes being the most important predictors (Fig 5b). We found a positive correlation between the abundances of chiggers and water depth (S = 4812, p-value = 0.000, rho = 0.2719), with host individuals with high chigger burdens tending to be from marshes with higher water depths.

**Table 2:** Rodent hosts of ectoparasites and landscape characteristics of 11 marshes in Tecopa, California, US between 2011 and 2019.

Marsh	Number of parasitized rodent individuals	Host species richness	Plant species richness	Water depth (cm)	Marsh area (m^2^)	Marsh perimeter (m)	Distance to nearest marsh (m)	Distance to nearest town (m)
1	45	2	5	0.404	18029	1170	15	674.4
7	33	1	10	1.017	1773	486	16	281.1
8	24	4	15	0.368	5481	744	290	415.9
9	21	4	13	2.006	7500	512	130	495.3
10	37	3	12	0.15	931	354	16	293.1
12	20	2	11	1.52	4116	622	14	487.6
17_21	29	3	10	1.675	26024	2300	86	105.9
39	34	3	11	0.5	5507	1006	14	421.9
54	66	2	4	4.61	9359	826	15	642.4
69	20	2	11	0.719	5	20	245	641.8
23	12	1	8	2.125	19950	1622	312	1101.75

## Discussion

Drivers of the composition of ecological communities of ectoparasites have important implications for animal and human health [12]. Here, we show that the primary determinants of ectoparasite community structure in our study system are host species and patch size, with host species richness in the community and other environmental factors such as patch distance, and water and plant availability not having significant influences. The influence of host species on structuring ectoparasite community could be explained by multiple combinations of phylogenetic, ecological, and geographical host-associated factors [69]. However, host phylogeny signal appears to be low: the ectoparasite community of the harvest mouse tends to be more similar to that of the Amargosa vole (subfamily Arvicolinae), and less similar to the closely-related species (cactus mice and woodrats) that belong to subfamily Neotominae [70]. The host influence on the ectoparasite community is consistent with the combination of the habitat use selection of the host and the presence of host-specialist parasites. The similarity of communities of ectoparasite is consistent with the fact that the Amargosa vole is highly habitat-specialized in Mojave Desert wetlands, in sympatry with the western harvest mouse and the alien house mouse [71]. On the other hand, cactus mice tend to live in drier areas, while woodrats in this site prefer mesquite shrublands. This is similar to findings in work in Israel that found that the structure of flea communities in a particular habitat was explained by host species composition and environmental factors [72].

The relatively depauperate flea fauna in the Amargosa study region featured only *O*. *sexdentatus* and *O*. *leucopus*, consistent with environment being harsh and wetlands being so isolated. Both species have strong, particular host affiliations, specifically woodrats for *O*. *sexdentatus* and *Peromyscus* species for *O*. *leucopus*, as occurred in the present study [49]. In the literature, *O*. *leucopus* is considered the most common flea of eastern white footed mice (*P*. *leucopus*) but “something of a rarity” in western North America [73–75]. It is also reported, although rarely, from hosts in the genera *Microtus*, *Blarina*, *Glaucomys*, *Ondatra*, *Clethrionomys*, *Tamias*, *Tamiasciurus*, *Reithrodontomys*, *Onychomys*, and *Sorex* [49,74]; an exception is a study in New Mexico which found extensive infestation of this flea on both *Peromyscus* spp. and *N*. *microplus* [76]. Most of the multiple subspecies of *O*. *sexdentatus* are woodrat fleas [49]. A study in Monterey County found more than 1000 *O*. *sexdentatus* on woodrats, and one or only a few on *P*. *californicus*, *P*. *maniculatus*, *M*. *californicus*, and a relatively large number of carnivores (possibly contaminated from woodrat prey or nest sites) [77]. Beyond host affiliations, little ecological information has been published for these species.

Similar to the fleas, identified ticks were limited to two species. *Ixodes mojavensis* was found on several host species, but partial host specificity for *M*. *californicus* is suggested by the finding that it did not occur in marshes where voles were not present. However, *I*. *mojavensis* is a newly discovered species, and its full geographic and host extent remains to be described [52]. *Ixodes mojavensis* was more abundant than *D*. *similis* across all marshes and hosts. In contrast with *I*. *mojavensis*, *D*. *similis* (formerly the western clade of *D*. *variabilis*, [68]) is a generalist, parasitizing a wide variety of hosts, with larvae and nymphs typically found on small mammals, and dogs often hosting the adults [78]. In this study, it was only found on harvest mice and voles. The presence of *D*. *similis* in the Amargosa is surprising, given that the surrounding desert climate should be inhospitable to the species and its presence is inconsistent with what is known about the distribution of the species in North America [79,80], suggesting possible introduction on pet dogs and that the marshes then provided a suitable microclimate with available small mammals to support immature tick stages. *Dermacentor variabilis* and *D*. *similis* have not previously been identified in desert environments. However, with the recent re-description of the western tick as the novel species *D*. *similis*, it is possible that the niche of this species is broader than was previously known.

Species richness of mites was somewhat greater than fleas and ticks, with two novel species not previously described. These included *Blankaartia* sp. n. which was found on voles, harvest mice, and house mice (previously reported in the Amargosa as *Neotrombicula microti*), and *Euschoengastia* putative sp. n. found on voles. We also found *Eutrombicula alfreddugesi*, but it was very rare and only on a house mouse. *Blankaartia* was the chigger most frequently identified on vole and could be identified to genus on the basis of: three σ on Ge I, the striated ornaments with punctuation of the Coxal field I-III, and the heart-shaped prodorsal sclerite which is characteristic of *Blankaartia*. This genus is described from the southern US, West Indies, Panama, and northern South America [56] but this appears to be the first time *Blankaartia* is reported from California. *Blankaartia* principally infests birds, in some cases associated with clinical trombiculiasis [81,82]. In Southeastern Brazil, *B*. *sinnamaryi* was found infected with a *Rickettsia felis*-like bacterium [83].

The second chigger, *E*. *alfreddugesi*, has also not previously been recorded in California, although it has been reported at least once before on house mice in Kansas [84]. It is the most frequently reported agent of human trombiculiasis in North America [55] but the literature is rife with misidentifications and there may be a complex of closely related, morphologically similar *Eutrombicula* responsible (reviewed in [85]). In fact, the species has been proposed as synonymous with *E*. *cinnabaris* which does occur in California [85]. The genus of *Euschoengastia* is globally distributed, with hosts including a variety of small mammals including *Microtus pennsylvanicus* [86] in particularly wet areas and (rarely) birds or lizards [87]. Numerous species are found from all over California, including several on *Microtus californicus* [88]. *Goffacarus latchmani* caused mange-like dermatitis on horses, dogs, and hares in California [89,90], while *E*. *xerothermobia* is associated with human trombiculiasis in Europe, and in Korea *E*. *koreaensis* is a vector of scrub typhus [91].

The Amargosa rodents that were most heavily infested with chigger mites were harvest mice, house mice, and the critically endangered Amargosa vole. Among chiggers, only the larval stage is parasitic, while other active life stages (nymph, adult) are typically predatory on other arthropods and arthropod eggs [92]. Chiggers are commonly seasonal- in Nebraska and Kansas, larval *E*. *alfreddugesi* appeared in April to May, peaked in abundance in late June and early July, and disappeared in mid-autumn as the ground began to freeze [84], while *Euschoengastia* appeared to experience population spikes following heavy rainfall events [87].

The other Amargosa mite found in our study is *Ornithonyssus bacoti*, a pest of veterinary importance which will also feed on humans as well. The tropical rat mite ingests host blood, can exsanguinate small mammals, and carries numerous pathogens naturally or following experimental infection [93–100]. Ecologically, it has a rapid developmental cycle, high fecundity, and prolonged off-host survival; unusually for a parasitic mite, it is able to travel a hundred meters to find a host [101]. An earlier study documented tropical rat mites on Amargosa voles in a captive colony, thought at the time to have been infested through contaminated straw exposed to mice near the facility [102], but the finding of the mite in nature suggests it may have been imported from founder individuals captured in the wild. Other voles from the Sierra Nevada were documented as hosts for this mite previously [103].

Abiotic drivers can be important factors in ectoparasite community structure, for example, rainfall and water sources which can affect abundance, developmental rates, and survival of ectoparasites and their hosts [30,31], particularly since most of the ectoparasites we found spend considerable time off the host. Even though in our study water depth was not significantly associated with variation in ectoparasite community composition, individual hosts had a higher number of chiggers in marshes with higher mean water depth. In contrast, distance to sites with people and other marshes did not emerge as important determinants. Habitat where chiggers thrive is reported to have high relative humidity, moderate temperature, and low incident sunlight [87,104]; fleas and ticks typically have humidity requirements specific to each species as well. Further study would be needed to document how broad is the niche for the novel tick, mites, and previously poorly studied fleas we observed.

Island biogeography theory predicts decreasing species richness in smaller and more isolated habitat patches [45,105]. While this theory was developed originally for patterns of species richness on actual islands at sea, numerous studies have applied it in terrestrial ecosystems e.g. [106–108] and host-parasite systems [109]. In our study, patch size was an important driver of the structure of the ectoparasite community, with Amargosa voles and harvest mice tending to have more trombiculid mites in the biggest patches and more *I*. *mojavensis* ticks in smaller patches. Patch size also influenced flea abundance and richness, likely through impacts on host habitat use, flea-host specificity patterns, and interspecific interactions. Southern marshes tended to be medium-sized patches surrounded by a more diverse plant community, and with multiple species of abundant rodents such as cactus mice and woodrats, compared with northern and central marshes. *Orchopeas* fleas predominated in these southern marshes, which is explained by the host-specificity of these flea species for cactus mice and woodrats [49]. These flea species tended to be in low numbers or absent in bigger patches where harvest mice and Amargosa voles predominated.

Interspecific interactions among ectoparasites—either positive (facilitation) or negative (competition)—could also be an important driver of an ectoparasite community [110]. Multiple mite taxa can often coexist on individual hosts [87], and we found at least 16 Amargosa voles hosting both chiggers and tropical rat mites. Generally however, our results showing significant negative relationships among ectoparasites on rodents suggests competitive exclusion interactions. Specifically, we found that individual Amargosa voles or harvest mice infested with mites were significantly less likely to host *I*. *mojavensis* ticks, and that tick-infested individuals had less mite infestation as well. This apparent competitive exclusion among ectoparasites may be due to physical or chemical communication (direct interaction) or resource exploitation (indirect interaction). Species with similar ecologies would tend to compete more [111], including anatomical preferences. Fleas and adult mites can range over the entire animal’s body, whereas ticks and chiggers often prefer to attach and feed near the head and ears, although chiggers have been reported to partition niches on individual hosts [86,87,103]. Facilitative and competitive interactions have been mainly studied in endoparasites and less so in ectoparasites [20–22], with exceptions including other studies in rodents which found apparent competitive exclusion among chiggers, ticks, fleas, and lice [23,24]; among fleas, chiggers, and cuterebrid botflies [25]; and among different flea species [26,27]. We considered whether our findings in the Amargosa could represent some form of confounding if for example mites tended to prefer one host species which is restricted to particular marshes while ticks favored a different host and marsh. However, this seems unlikely given that voles and harvest mice were preferred hosts for both *I*. *mojavensis* and chiggers, supporting true competition. Also, it is interesting that chiggers are often in negative interactions among ectoparasites in our data and in the literature, but the underlying mechanisms are unknown. Chiggers secrete digestive enzymes into host skin to create a feeding structure called a stylostome [112]; perhaps host reactions to the salivary secretions reduce favorability for other ectoparasite species. Another possibility could be that one parasite affects host activity, e.g. through inducing weight loss or lethargy, and this reduces movement and encounters with other parasites. Further experimental studies could help identify mechanisms underlying the competitive exclusion among ectoparasites of Mojave Desert rodents.

Some of the ectoparasites found in this study may be vectors of diseases of conservation or medical concern. Despite that chigger infestation is relatively common in some rodents, it is rarely associated with disease with the exception of scrub typhus, an important disease of people in parts of Asia that is transmitted by chiggers [103,113,114]. For instance, Amargosa voles experience severe and highly prevalent lesions due to chiggers [115], while California voles (the larger species of which Amargosa voles are a subspecies) have been shown to be fatally susceptible to plague and tularemia [6,116]. Chiggers on Amargosa voles are highly prevalent, and often associated with severe lesions, although their presence did not appear to impact body mass or fitness [117]. The fleas *O*. *sexdentatus* and *O*. *leucopus* have been found naturally infected with *Yersinia pestis*, the causative agent of plague [118]. Plague represents a serious concern for conservation efforts of some rodent species, such as prairie dogs (*Cynomys* spp.) [119]. *Dermacentor variabilis* ticks have been reported infected with other *Francisella tularensis*, the causative agent of tularemia [120]. *Ixodes* species ticks are vectors of *Borrelia burgdorferi* sensu lato group, some of which are associated with Lyme disease. *Ixodes mojavensis* and Amargosa voles have been found to carry *Borrelia carolinensis*, a bacterium of unknown pathogenicity [121]. Our group has conducted several disease surveys among Amargosa rodents [122–124], but continued vigilance is essential both for public health as well as protection of endangered species.

There were a few important limitations to the study, for example, that not all mites were identified to species, as other authors have done for small mammals where mite infestation is abundant [103]. In addition, it appears that several of the mites we observed may be novel species, such that data on host preference and other aspects of their ecology are not yet available. Given how small many of the parasites studied are and that animals were examined alive in the field, we could not be completely confident in ruling out low burden infestations and were concerned about misclassification bias in classifying animals as uninfested, which was why we elected not to analyze for infestation prevalence and instead focused on relative abundance. Our use of relative abundance also allowed for use of statistical methods that account for clustering within the data.

We provide insights into ectoparasite ecology and host-parasite interactions in a very remote and isolated marshy habitat supporting multiple endangered species, where ongoing habitat degradation and loss of water driven by anthropogenic hydrologic alterations (e.g. ground-water pumping and land clearing) and climate change have considerable impacts on ecological interactions. Knowledge of ectoparasite composition and ectoparasite infestation of rodents in this region is important not only because it identifies the potential ectoparasite vectors, but it also provides information needed to design and implement programs to manage vector-borne diseases for purposes of wildlife conservation and human health.

## Supporting information

S1 Table Ectoparasite and landscape data used in analysis. (XLS)
